# Limited clinical utility for GWAS or polygenic risk score for postoperative acute kidney injury in non-cardiac surgery in European-ancestry patients

**DOI:** 10.1186/s12882-022-02964-8

**Published:** 2022-10-21

**Authors:** Daniel B. Larach, Adam Lewis, Lisa Bastarache, Anita Pandit, Jing He, Anik Sinha, Nicholas J. Douville, Michael Heung, Michael R. Mathis, Jonathan D. Mosley, Jonathan P. Wanderer, Sachin Kheterpal, Matthew Zawistowski, Chad M. Brummett, Edward D. Siew, Cassianne Robinson-Cohen, Miklos D. Kertai

**Affiliations:** 1 Department of Anesthesiology, Vanderbilt University Medical Center, Nashville, TN USA; 2 Department of Biomedical Informatics, Vanderbilt University Medical Center, Nashville, TN USA; 3 Department of Biostatistics, University of Michigan, Ann Arbor, MI USA; 4 Department of Anesthesiology, University of Michigan, Ann Arbor, MI USA; 5 Institute of Healthcare Policy & Innovation, University of Michigan, Ann Arbor, MI USA; 6 Division of Nephrology, Department of Internal Medicine, University of Michigan, Ann Arbor, MI USA; 7 Department of Computational Medicine and Bioinformatics, University of Michigan, Ann Arbor, MI USA; 8 Department of Medicine, Vanderbilt University Medical Center, Nashville, TN USA; 9 Division of Nephrology and Hypertension, Vanderbilt Center for Kidney Disease (VCKD) and Integrated Program for AKI (VIP-AKI), Tennessee Valley Health System, Nashville Veterans Affairs Hospital, Nashville, TN USA; 10 Vanderbilt O’Brien Kidney Center, Division of Nephrology and Hypertension, Department of Medicine, Vanderbilt University Medical Center, Nashville, TN USA; 11 Division of Adult Cardiothoracic Anesthesiology, Department of Anesthesiology, Vanderbilt University Medical Center, 1211 21st Avenue South, Medical Arts Building, Office 526E, Nashville, TN 37212 USA

**Keywords:** Acute kidney injury, Genome-wide association study, Polygenic risk score, Surgery

## Abstract

**Background:**

Prior studies support a genetic basis for postoperative acute kidney injury (AKI). We conducted a genome-wide association study (GWAS), assessed the clinical utility of a polygenic risk score (PRS), and estimated the heritable component of AKI in patients who underwent noncardiac surgery.

**Methods:**

We performed a retrospective large-scale genome-wide association study followed by a meta-analysis of patients who underwent noncardiac surgery at the Vanderbilt University Medical Center (“Vanderbilt” cohort) or Michigan Medicine, the academic medical center of the University of Michigan (“Michigan” cohort). In the Vanderbilt cohort, the relationship between polygenic risk score for estimated glomerular filtration rate and postoperative AKI was also tested to explore the predictive power of aggregating multiple common genetic variants associated with AKI risk. Similarly, in the Vanderbilt cohort genome-wide complex trait analysis was used to estimate the heritable component of AKI due to common genetic variants.

**Results:**

The study population included 8248 adults in the Vanderbilt cohort (mean [SD] 58.05 [15.23] years, 50.2% men) and 5998 adults in Michigan cohort (56.24 [14.76] years, 49% men). Incident postoperative AKI events occurred in 959 patients (11.6%) and in 277 patients (4.6%), respectively. No loci met genome-wide significance in the GWAS and meta-analysis. PRS for estimated glomerular filtration rate explained a very small percentage of variance in rates of postoperative AKI and was not significantly associated with AKI (odds ratio 1.050 per 1 SD increase in polygenic risk score [95% CI, 0.971–1.134]). The estimated heritability among common variants for AKI was 4.5% (SE = 4.5%) suggesting low heritability.

**Conclusion:**

The findings of this study indicate that common genetic variation minimally contributes to postoperative AKI after noncardiac surgery, and likely has little clinical utility for identifying high-risk patients.

**Supplementary Information:**

The online version contains supplementary material available at 10.1186/s12882-022-02964-8.

## Introduction

Acute kidney injury (AKI) occurs in up to 20% of hospitalized adults [1–3], with higher incidence rates in older individuals [4]. Patients in the perioperative setting are at elevated risk for AKI, with a reported overall postoperative incidence of 7.4% [5]. Those undergoing major surgery are considerably more susceptible to AKI with incidence rates ranging from 13 to 32% depending upon the type of surgery [6–9]. Of great significance, overall hospital mortality rates in the setting of AKI approach 25%, with mortality exceeding 50% when renal replacement therapy is necessary [3, 10–13].

At present, clinical management of AKI is chiefly supportive, as no proven therapies exist to reverse or reduce the severity of its course. As such, prevention is the mainstay of avoiding the health consequences of AKI. The Kidney Disease: Improving Global Outcomes (KDIGO) Clinical Practice Guideline for Acute Kidney Injury identifies the need for AKI risk prediction tools [14]. Some clinical risk factors are well-established, including older age, diabetes mellitus, pre-existing chronic kidney disease (CKD), and congestive heart failure. While risk prediction tools exist [15–18], none have been brought into widespread clinical implementation. Identifying a broad set of risk factors and mechanisms contributing to AKI is critical to identifying high-risk patients and developing mitigation strategies to decrease postoperative AKI risk.

Large genome-wide association studies (GWAS) have identified multiple single nucleotide polymorphisms (SNPs) associated with baseline renal function and CKD, confirming that common genetic variation modulates kidney physiology [19, 20]. However, to date, only hypothesis-driven candidate gene studies and small GWAS of hospitalized patients [21] and those undergoing cardiac surgery [22, 23] have investigated the contribution of common genetic variation to AKI risk [24]. To address these knowledge gaps, we conducted a large-scale GWAS of genotyped patients who underwent noncardiac surgery at 2 quaternary academic medical centers to identify novel genetic mechanisms contributing to postoperative AKI.

## Methods

The study design and data reporting followed the recommendations of Strengthening the Reporting of Genetic Association Studies (STREGA) [25]. We used 2 independent cohorts of patients who underwent noncardiac surgery at Vanderbilt University Medical Center (“Vanderbilt”; Nashville, TN, USA) and Michigan Medicine of the University of Michigan (“Michigan”; Ann Arbor, MI, USA) to conduct initial common variant discovery by GWAS followed by meta-analysis of top candidate SNPs. The study was approved by the Vanderbilt University Institutional Review Board and the Institutional Review Boards of the University of Michigan Medical School. Given the retrospective design of the study and the use of deidentified data, both boards waived the need for informed patient consent.

Eligible patients were 18 years and older, enrolled in the Multicenter Perioperative Outcomes Group (MPOG) database, had a baseline creatinine value within 365 days before to surgery, had one or more creatinine values recorded within the first 7 days after surgery (including postoperative day 0), and had site-specific genotype data that passed quality control. Subsequently, anesthesia base Current Procedural Terminology (CPT) codes were used to identify patients who underwent noncardiac surgery. Patients with CPT codes for the following cases were excluded from the current study: cardiac surgeries and liver transplants (due to their high acuity and intraoperative heterogeneity), kidney transplants and urologic kidney/ureter surgeries (due to the likelihood of surgical manipulation leading to postoperative creatinine changes), and obstetric non-operative procedures, pain medicine procedures, and electroconvulsive therapy. Similarly, patients were excluded if they 1) underwent surgery classified as outpatient or 23-hour-admission; 2) were missing primary anesthesia base CPT code data; 3) had preoperative chronic renal failure (eGFR < 15 mL/min/1.73 m^2^); 4) were dependent on hemodialysis (based on the presence of prior anesthesia CPT code for dialysis access or International Classification of Diseases [ICD] codes for end-stage renal disease); 5) had preoperative AKI during the 7 days before to surgery (relative serum creatinine increase of > 50% or absolute serum creatinine increase of > 0.3 mg/dL); 6) were classified as American Society of Anesthesiologists physical status VI; 7) had a procedure with a duration < 45 minutes; 8) had a subsequent surgery within 7 days without documentation of a postoperative creatinine between cases; or 9) had implausible body mass index values (< 10 or > 80 kg/m^2^). For patients with more than one eligible surgical case, only the first case was included.

**Fig. 1:**
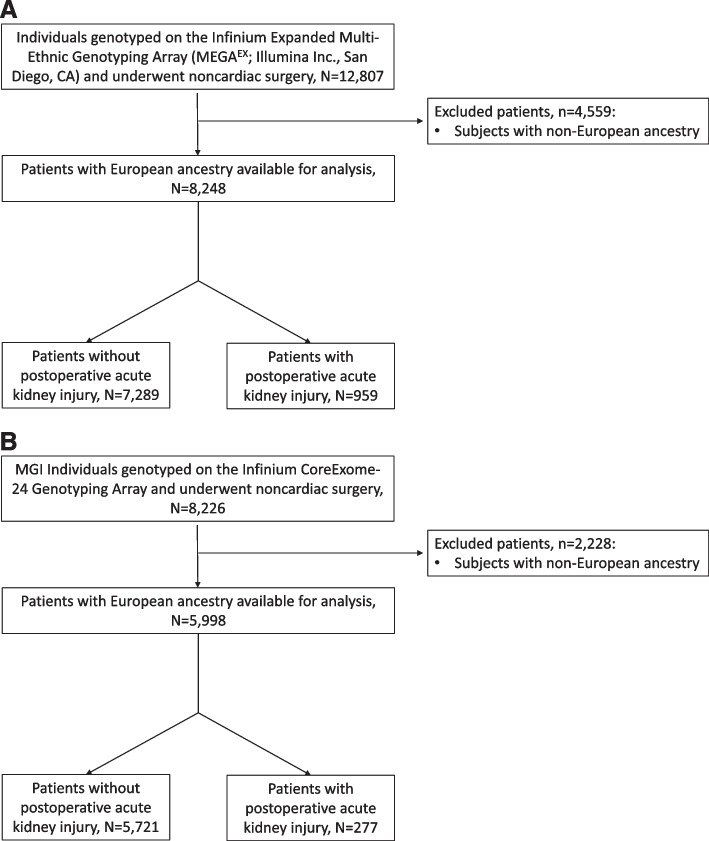
Flowchart of the Vanderbilt (**A**) and Michigan (**B**) study populations with inclusion and exclusion criteria

The Vanderbilt Department of Anesthesiology is one of the funding and participating centers of the MPOG. In brief, data on unique perioperative records spanning from 2008 to the present day are collected by the MPOG participating sites and integrated within a secure database at the University of Michigan on a regular monthly basis. The Vanderbilt cohort for GWAS was selected from the MPOG database and linked with Vanderbilt’s DNA biobank (BioVU) for available genetic information. This cohort of patients consisted of 8248 adults of who met our inclusion criteria, were of White European ancestry, and who underwent noncardiac surgery at Vanderbilt between 2008 and 2017 and had extant genotyping data as part of an institutional initiative to genotype a large, diverse patient population (Fig. 1). Given the small number of patients with ancestry other than White European ancestry in the Vanderbilt cohort these patients were excluded from the present study.

The Michigan cohort was also selected from the MPOG database and linked to the Michigan Genomics Initiative (MGI), a longitudinal biorepository within Michigan Medicine with genetic data linkable to medical phenotype and electronic health record information. The cohort consisted of 5998 adults of European ancestry who met our inclusion criteria and underwent noncardiac surgery at Michigan between 2012 and 2017 and had extant genotyping data in the MGI database (Fig. 1). Similarly to the Vanderbilt cohort, given the small number of patients with ancestry other than White European in the Michigan cohort these patients were excluded from the present study.

### Clinical data and outcome definition

Patient demographics (age, sex), preoperative characteristics (BMI, creatinine, hemoglobin, and Elixhauser comorbidity measures [26]) and procedural factors (case duration and type of anesthesia performed) that have been previously identified as risk factors for postoperative AKI [18] were also extracted from the participating sites’ respective institutional MPOG databases.

The outcome of the current study was postoperative AKI, ascertained according to the KDIGO Clinical Practice Guideline for Acute Kidney Injury with modifications because information on urine output was absent [14]. Using previously developed methodology [27], we defined postoperative AKI using the most recent preoperative (baseline) serum creatinine values that were collected up to 365 days before surgery and all available serum creatinine values that were measured within 7 days after surgery or until discharge, whichever came first. The diagnosis of postoperative AKI was made if there was a 50% or greater serum creatinine increase in the first 7 postoperative days, or a 0.3 mg/dL increase detected using a rolling 54-hour window starting at anesthesia end time and ending 7 days later in the postoperative period. The 54-hour rolling window was selected to add 3 hours’ buffer on each side of the usual 48-hour window to account for minor variations in serum creatinine blood draw and laboratory reporting times. Missing data was excluded from analysis where applicable, which only occurred in the comprehensive analyses.

### Genetic data

The Vanderbilt cohort subjects were genotyped using DNA extracted from discarded plasma samples with the Illumina Infinium® Expanded Multi-Ethnic Genotyping Array (MEGA^EX^, Illumina Inc., San Diego, CA, USA). Michigan cohort subjects were genotyped using Illumina Infinium® CoreExome Array (Illumina Inc., San Diego, CA, USA) [28, 29]. Additional methods on quality control and imputation are available in the data supplement.

### Glomerular filtration rate GWAS summary statistics and polygenic risk score

Summary statistics for baseline renal function as well as a polygenic risk score (PRS) for decreased estimated glomerular filtration rate (eGFR) were obtained from a large meta-analysis of GWAS of eGFR [19].

### Statistical analysis

Descriptive statistics of clinical variables are presented as frequency and percentage for categorical variables and median [interquartile range] for continuous variables.

For each of the SNPs in the Vanderbilt and Michigan cohorts as part of the GWAS, allelic associations with postoperative AKI were assessed using logistic regression analyses adjusted for our prespecified patient demographics, preoperative and procedural factors. In the primary analyses (model I), we adjusted for age, gender and 4 principal components (PCs). Exploratory analyses adjusted for: (model II) age, gender, 4 PCs and log-transformed preoperative serum creatinine; or (model III) for age, gender, 4 PCs, preoperative (body mass index, creatinine, hemoglobin, Elixhauser comorbidity measures), and procedural factors (case duration, general anesthesia performed). Association analyses for Vanderbilt were conducted using PLINK (https://www.cog-genomics.org/plink/1.9/) for all genotyped and imputed markers assuming an additive inheritance model (homozygote major allele vs heterozygote vs homozygote minor allele). Association analyses for Michigan were conducted using SAIGE (https://www.nature.com/articles/s41588-018-0184-y). Markers were considered to be genome-wide significant if *P* < 5 × 10^− 8^, which is the commonly used Bonferroni-adjusted significance threshold for GWAS.

We then conducted a meta-analysis using the effect size estimates and standard errors across studies, a classical approach implemented in METAL (http://csg.sph.umich.edu/abecasis/metal/) to assess the overall effect of candidate SNPs. The final candidate gene(s) were prioritized based on reaching statistical significance in the meta-analysis.

The previously published PRS [19] for eGFR was tested with the Vanderbilt cohort. A clumping algorithm was used to select a linkage-disequilibrium reduced set of SNPs (R^2^ < 0.05) with a minor allele frequency > 5% associated with postoperative AKI (*P* < 5 × 10^− 3^). The final score comprised 7949 SNPs. For each Vanderbilt individual, the PRS was computed by multiplying the SNP weighting by the allele dose for each SNP and summing up the products. The PRS represents genetically determined eGFR level an individual carries based their additive genetic risk for the 7949 SNPs.

The PRS was standardized to a mean of 0 and standard deviation of 1, so odds ratios represent the change in postoperative AKI risk per standard deviation change in the PRS. Linear regression models were used to test the association between the eGFR PRS and preoperative Cr, controlling for age, gender, BMI, and 4 PCs. A logistic regression was used to assess the association between the eGFR PRS and postoperative AKI, controlling for covariates specified in model III.

We used genetic mixed models, as implemented in the Genome-wide Complex Trait Analysis (GCTA) pack [30] to estimate the additive SNP heritability of postoperative AKI in the Vanderbilt cohort. Heritability was defined as the proportion of postoperative AKI liability in the Vanderbilt cohort attributable to additive common genetic variation. The GCTA method uses a mixed model in conjunction with a genetic relationship matrix (GRM) of pairs of samples based on 840,964 SNPs to estimate the proportion of variance explained by genetic similarity between individuals. GCTA provides a lower limit of the variance that can be theoretically explained by common SNPs. In our study, the estimated heritability was transformed from a liability scale to an observed scale assuming the incidence of postoperative AKI was 4.5%. The GCTA analyses were adjusted for age, gender and 4 PCs.

All statistics tests were 2-sided. A *P*-value < 0.05 was considered statistically significant, unless otherwise noted. Analyses were performed using R version 4.0.0. unless stated otherwise.

## Results

The study consisted of 8248 patients in the Vanderbilt cohort and 5998 patients in the Michigan cohort. Demographics and clinical characteristics of the patients in these 2 cohorts, stratified according to the documented presence or absence of postoperative AKI, are shown in Table 1. The mean age was 58.05 ± 15.23 years in the Vanderbilt cohort and 56.24 ± 14.76 years in the Michigan cohort. Both cohorts had similar proportions of male patients (4139 [50.2%] in the Vanderbilt cohort and 2942 [49%] in the Michigan cohort). Postoperative AKI was relatively common and occurred at a higher rate in the Vanderbilt cohort (959 [11.6%], of which 78.5% [*n* = 753], 15.7% [*n* = 151], 5.8% [*n* = 56] met criteria for stages I, II, and III AKI) compared with the Michigan cohort (4.6% [*n* = 277] [4.6%], of which 81% [*n* = 224], 14% [*n* = 39], 5% [*n* = 14] met criteria for stages I, II, and III AKI). Patients with postoperative AKI in both cohorts had a higher frequency of several relevant patient- and procedure-related characteristics compared with patients without postoperative AKI in both study populations (Table 1).

**Table 1:** Baseline and clinical characteristics of the noncardiac surgery study population

	Vanderbilt Cohort			Michigan Cohort		
Characteristics	Yes AKI, ***n*** = 959	No AKI, ***n*** = 7289	***P*** value	Yes AKI, n = 277	No AKI, ***n*** = 5721	***P*** value
Age, years	61.6 [52.3–70.1]	59.3 [48.3–68.6]	1.33 × 10^− 4^	61.0 [51.0–69.0]	58.0 [47.0–67.0]	4.63 × 10^− 4^
Male sex	571 (59.5%)	3568 (48.9%)	4.34 × 10^− 7^	152 (54.9%)	2790 (48.8%)	0.049
Body mass index, kg/m^2^	29.43 [25.3–34.6]	27.69 [23.88–32.52]	9.99 × 10^−11^	29.91 [25.95–36.6]	29.48 [25.7–34.6]	0.495
Preoperative creatinine value, mg/dL	0.97 [0.77–1.26]	0.90 [0.74–1.09]	8.25 × 10^−4^	0.89 [0.7–1.1]	0.83 [0.71–0.99]	0.019
Preoperative hemoglobin, mg/dL	12.6 [10.8–14.0]	13.2 [11.8–14.4]	3.71 × 10^−13^	12.55 [10.7–14.2]	13.60 [12.5–14.7]	3.21 × 10^− 12^
Case duration, min	205 [128.5–289.5]	165 [106–251]	1.26 × 10^− 10^	285.0 [193.0–389.0]	199.0 [136.0–305.0]	2.2 × 10^− 16^
General anesthesia	911 (95%)	6658 (91.3%)	2.87 × 10^−3^	274 (98.9%)	5163 (90.2%)	8.43 × 10^−9^
Elixhauser comorbidities						
AIDS/HIV	3 (0.31%)	10 (0.14%)	0.197	0 (0%)	8 (0.14%)	1
Alcohol abuse	18 (1.9%)	124 (1.7%)	0.694	8 (2.9%)	10 (1.9%)	0.262
Blood loss anemia	6 (0.63%)	24 (0.3%)	0.152	3 (1.1%)	38 (0.7%)	0.437
Chronic pulmonary disease	134 (14%)	768 (10.5%)	0.001	63 (22.7%)	1073 (18.8%)	0.099
Congestive heart failure	95 (10%)	432 (4.7%)	8.04 × 10^−12^	26 (9.4%)	243 (4.2%)	2.81 × 10^−4^
Deficiency anemia	11 (1.5%)	38 (1%)	0.192	15 (5.4%)	110 (1.9%)	5.85 × 10^−4^
Diabetes mellitus						
Uncomplicated	123 (12.8%)	694 (9.5%)	0.001	66 (23.8%)	898 (15.7%)	5.69 × 10–4
Complicated	17 (1.1%)	52 (0.5%)	0.018	14 (5.1%)	125 (2.2%)	0.004
Hypertension						
Uncomplicated	298 (28.6%)	2085 (31.1%)	0.113	147 (53.1%)	2666 (46.6%)	0.036
Complicated	61 (1.9%)	158 (0.7%)	2.85 × 10^−14^	48 (17.3%)	200 (3.5%)	2.2 × 10^−16^
Liver disease	70 (7.3%)	234 (3.2%)	2.54 × 10^−10^	35 (12.6%)	426 (7.4%)	0.004
Metastatic cancer	67 (7%)	525 (7.2%)	0.807	65 (23.5%)	807 (14.1%)	5.15 × 10^−5^
Peripheral vascular disorders	55 (5.7%)	322 (4.4%)	0.066	45 (16.2%)	469 (8.2%)	2.18 × 10^− 5^
Pulmonary circulation disorders	34 (3.5%)	120 (1.6%)	4.39 × 10^−5^	19 (6.9%)	149 (2.6%)	2.43 × 10^−4^
Rheumatoid arthritis/collagen vascular disease	20 (2.1%)	173 (2.4%)	0.579	8 (2.9%)	230 (4.0%)	0.431
Valvular disease	71 (7.4%)	323 (4.4%)	4.94 × 10^−5^	16 (5.8%)	177 (3.1%)	0.021

### Genome-wide association results

In the primary analyses, there were no SNPs associated with AKI that reached genome-wide significance. However, 5 SNPs met a suggestive threshold of *P* < 5*10^− 6^. These SNPs were subsequently analyzed in a series of logistic regression models, conducted as part of the meta-analysis of both data sets. The meta-analyses of the genome wide association results are depicted using Manhattan plots (Supplementary Fig. 1) and quantile-quantile plots, which showed good adherence to null expectations (Supplementary Fig. 2).

In the first analysis after adjusting for age, gender and the 4 PCs, a single SNP (rs975593) on chromosome 21 with meta-*P* = 6.62*10^− 7^ and OR = 1.35 (SE = 1.06) was associated with postoperative AKI in both cohorts (Table 2). When the analyses were further adjusted for preoperative serum creatinine (second analysis) 2 additional SNPs were identified. The SNP (rs2255595) on chromosome 10 was associated with a reduced risk for postoperative AKI in both cohorts, but this association did not reach genome-wide significance (meta-*P* = 8.62 × 10^− 7^ and OR = 0.80 (SE = 1.04)). The other SNP (rs143469518) identified on chromosome 17 was associated with a higher risk for postoperative AKI in both cohorts, but again this association did not reach genome-wide significance (meta-*P* = 8.46*10^− 7^ and OR = 1.77 [SE = 1.12]). Finally, in the third analysis with a fully adjusted model that also included patient-, clinical-, and procedure-related risk factors, 3 SNPs on chromosome 17 showed association with postoperative AKI. The SNP rs143469518 remained associated (meta-*P* = 2.16*10^− 7^ and OR = 1.93 [SE = 1.13]) with a higher risk for postoperative AKI, and 2 additional SNPs were associated with a reduced risk for postoperative AKI without reaching genome-wide significance (rs2069295, meta-*P* = 4.84*10^− 7^; and rs117284771, meta-*P* = 3.69*10^− 7^) (Table 2).

**Table 2:** Logistic regression analysis of genetic predictors of postoperative acute kidney injury in the noncardiac surgery study population

				Vanderbilt Cohort				Michigan Cohort						Combined
				TAF				TAF						
Chr	SNP	POS	Gene symbol	No AKI	Yes AKI	OR (95% CI)	P	No AKI	Yes AKI	OR (95% CI)	P	Meta-OR	SE (OR)	Meta-P
**MODEL I: adjusted for age, gender, 4 principal components**														
21	rs975593	24,965,000	D21S2088E | LINC01689	0.11	0.15	1.38 (1.20–1.59)	4.80 × 10^−6^	0.12	0.15	1.28 (1.01–1.62)	0.042	1.35	1.06	6.62 × 10^−7^
**MODEL II: adjusted for age, gender, 4 principal components, and preoperative serum creatinine**														
10	rs2255595	1,455,543	ADARB2	0.57	0.53	0.81 (0.73–0.89)	5.16 × 10^−5^	0.59	0.53	0.80 (0.68–0.93)	0.005	0.80	1.05	8.62 × 10^−7^
17	rs143469518	46,040,327	PRR15L | CDK5RAP3	0.02	0.04	1.88 (1.46–2.44)	1.51 × 10^−6^	0.03	0.04	1.43 (0.88–2.30)	0.145	1.77	1.12	8.46 × 10^−7^
21	rs975593	24,965,000	D21S2088E | LINC01689	0.11	0.14	1.38 (1.20–1.59)	5.26 × 10^−6^	0.12	0.15	1.28 (1.01–1.62)	0.041	1.36	1.06	7.11 × 10^−7^
**MODEL III: adjusted for age, gender, 4 principal components, preoperative serum creatinine, body mass index, preoperative hemoglobin, Elixhauser comorbidity measures, case duration, general anesthesia performed**														
17	rs2069295	45,995,891	SP2-AS1	0.98	0.96	0.49 (0.37–0.65)	9.40 × 10^−7^	0.97	0.96	0.67 (0.39–1.14)	0.138	0.53	1.14	4.84 × 10^−7^
17	rs117284771	46,053,767	CDK5RAP3	0.98	0.96	0.48 (0.36–0.63)	2.60 × 10^−7^	0.97	0.96	0.74 (0.43–1.25)	0.259	0.53	1.14	3.69 × 10^−7^
17	rs143469518	46,040,327	PRR15L | CDK5RAP3	0.02	0.04	2.09 (1.57–2.71)	3.54 × 10^−7^	0.03	0.04	1.49 (0.87–2.55)	0.143	1.94	1.14	2.16 × 10^−7^

*AKI* acute kidney injury, *Chr* chromosome, *CI* confidence interval, *Meta* meta-analysis, *OR* odds ratio, *POS* position, *SE* standard error, *SNP* single-nucleotide polymorphism, *TAF* test allele frequency

### Estimated glomerular filtration rate GWAS summary statistics and polygenic risk score

To ascertain whether genetic mechanisms associated with eGFR also contribute to AKI risk, we tested for an association with the previously published PRS for eGFR in the Vanderbilt cohort. The PRS was significantly associated with lower preoperative log-transformed creatinine level in our cohort, − 0.043 per 1 SD increase in PRS (95% CI, − 0.049 to − 0.036; *P* = 1.55*10^− 33^). This implies that 1 SD increase in PRS was associated with 4.2% lower serum creatinine level (95% CI, 3.5–4.8%). AKI cases, as compared with controls, had higher PRS for eGFR values. After adjusting for age, gender, preoperative serum creatinine, patient-, clinical-, and procedure-related risk factors of postoperative AKI, and 4 PCs, the PRS for eGFR was not significantly associated with postoperative AKI (0.049 per 1 SD increase in PRS; 95% CI, − 0.029-0.126; *P* = 0.22). This implies that 1 SD increase in PRS for eGFR after adjusting for other variables in the model was associated with a higher odds ratio of 1.05 (95% CI, 0.971–1.13) for postoperative AKI, but this association was not statistically significant.

### Genome-wide complex trait analysis results

We estimated the extent to which common variants account for AKI risk. The additive heritability estimate for postoperative AKI in the Vanderbilt cohort was 4.5% (SE = 4.5%), suggesting that there is a low contribution of common genetic variation to postoperative AKI in noncardiac surgery risk. In comparison, the heritability of preoperative serum creatinine in the Vanderbilt cohort was 9.8% (SE = 1.06%), supporting the validity of our renal function phenotypes and also indicating a much greater heritability component determining baseline kidney function.

## Discussion

In our study, we could not identify genetic variants that met the threshold for genome-wide statistical significance for predicting postoperative AKI following noncardiac surgery. Consistent with these findings, we found that a PRS for eGFR was not associated with postoperative AKI risk and offered no improvement in predicting the risk of postoperative AKI following noncardiac surgery. Furthermore, our heritability analysis also indicated a very low estimated heritability for postoperative AKI. Of note, we found that the PRS for eGFR in our study was significantly associated with elevated preoperative serum creatinine values indicating a stronger genetic predisposition. Thus, the findings of this study indicate that genetic variation minimally contributes to postoperative AKI after noncardiac surgery, and likely has little clinical utility for identifying high-risk patients.

None of the SNPs meta-analyzed across both institutions reached the commonly accepted genome-wide significance level of *P* < 5 × 10^− 8^. However, 5 SNPs did reach a significance level of P < 5 × 10^− 6^ in both cohorts (with identical directionality across cohorts for each variant). SNP rs975593 is located within a noncoding region on chromosome 21, in proximity to several pseudogenes and uncharacterized genes of uncertain significance. It is about 200 kilobases from the *D21S2088E* gene, which has been associated with subclinical interstitial lung disease [31]. An intron of chromosome 10 gene *ADARB2* (also known as *ADAR3*) contains variant rs2255595. This gene encodes the inactive RNA-editing enzyme adenosine deaminase RNA specific B2, which is primarily expressed in the brain and has been linked with learning and memory [32], longevity, and atherosclerosis risk factors [33], and systolic blood pressure [34]. Finally, the 3 near-significant chromosome 17 SNPs are located within 58 kilobases of each other and most likely represent the same genetic signal. Variant rs117284771 is located within an intron of *CDK5RAP3*, which encodes CDK5 regulatory subunit associated protein 3. Variation in this tumor suppressor has been linked with tumorigenesis and metastasis for several cancers including renal, hepatocellular, and gastric carcinomas [35–37]. It has also been associated with endothelial nitric oxide synthase activity and systolic blood pressure changes in mice [38]. SNP rs2069295 is located within an intron of nearby gene *SP2*, which encodes the Sp2 transcription factor. This protein has been linked with hepatocellular cancer [39]. The third chromosome 17 variant, rs143469518, is found near *CDK5RAP3* as well as *PRR15L* (encoding proline rich 15 like protein) and *PNPO* (encoding pyridoxamine 5′-phosphate oxidase). There is scant literature regarding *PRR15L*, while the protein encoded by *PNPO* catalyzes the terminal, rate-limiting step in vitamin B6 synthesis. *PNPO* mutations can cause a form of neonatal epileptic encephalopathy; although this gene is highly expressed in the kidney, the clinical syndrome does not appear to include renal abnormalities [40].

In addition to examining individual SNPs for association with AKI, we applied a previously published PRS for baseline renal function to one of our cohorts [19]. Although AKI cases had higher PRS scores than controls, this association did not meet statistical significance after adjusting for pertinent risk factors and did not improve discriminatory capability. Similarly, GCTA found a low heritability estimate for postoperative AKI (4.5%; SE = 4.5%), but a much higher heritability for preoperative baseline creatinine (9.8%; SE = 1.06%). Consequently, there is likely a much stronger genetic basis for baseline renal function and CKD compared with AKI. This accords with the known highly heritable nature of CKD, estimated to be between 30 and 75% [41].

In this study, we observed that many previously described risk factors were more prevalent in patients with postoperative AKI. Indeed, these observations provide further support for the concept of using a set of patient- and procedure-related risk factors--many of which are modifiable --to identify before surgery those high-risk noncardiac surgery patients who could benefit from risk management strategies aimed at mitigating the risk for postoperative AKI [42].

This study has potential limitations. First, despite being larger in size than previously published AKI GWAS, this study is powered to detect only common variants with relatively large effect sizes. Since only variants with MAF > 0.01 (1%) were assessed in the final meta-analysis, the possibility for rare genetic variants that drive a pronounced clinical phenotype was not explored. Larger samples, perhaps including sequence data, may help identify common AKI-associated variants with smaller effect sizes or very rare mutations that are undetectable with genotype data. Second, modifications of the accepted KDIGO definitions were required due to the absence of accurate urine output data or data on renal replacement therapy. Similarly, our cohorts were likely enriched for patients with AKI given that patients having low-risk surgeries may not have had postoperative creatinine values drawn (excluding them) and patients having outpatient surgeries were excluded altogether. This likely influenced our data on postoperative AKI incidence. Third, excluding patients with ancestry other than White European due to the relatively small number of these patients in the Vanderbilt and Michigan cohorts prevented us from studying the potential association of genetic predictors in the risk of postoperative AKI in these groups of noncardiac surgery patients. Therefore, future multi-institutional studies aiming at including patients with ancestry other than White European should be conducted. Fourth, applying a PRS for predicting baseline kidney function that was developed and validated in a general population of European -ancestry individuals to patients undergoing noncardiac surgery may not capture and characterize the genetic contribution to postoperative AKI. Thus, future studies are needed to characterize how such PRS could predict the risk for postoperative AKI. Finally, our AKI incidence (11.6% in the Vanderbilt cohort and 4.6% in the Michigan cohort) is lower than found in previous studies (13–32%) [6–9]. This difference likely results from difference in surgical type (for example, the incidence after major abdominal surgery was 13.4% [7] and after cardiopulmonary bypass was 18.2% [8]). Furthermore, a selection bias likely results from biobank enrollment, more likely after elective, non-emergent procedures scheduled during routine hours.

A final limitation is that even the full regression model (model III) fails to control for intraoperative confounders potentially contributing to the development of AKI. We took the approach of not including intraoperative factors, as these are downstream in the causal pathway from when a preoperative decision to pursue renoprotective interventions would be useful. If intraoperative risk factors are included, then the model is not clinically actionable until the postoperative period. Surgical duration was notably included in the preoperative risk score, with the assumption that for most cases, the approximate duration of the case would likely be known preoperatively (which of course has limitations, but on balance we decided was better to include in the preoperative risk score rather than leave out). Future studies may specifically assess for intraoperative associations, including potential nephrotoxic medication administered, the presence of intravenous or intra-arterial contrast, blood transfusion, emergency surgery, and procedural technique.

One recent study specifically assessed the influence of intraoperative hypotension, a modifiable clinical risk factor [27]. In this study, severe intraoperative hypotension (mean arterial pressure < 50 mmHg) was associated with postoperative AKI in medium-risk cohorts, while mild intraoperative hypotension (mean arterial pressure 55 to 59 mmHg) was associated in high-risk cohorts [27]. Despite these recent advances understanding the role of intraoperative hypotension on AKI, the lack of standardized definition further complicates straightforward incorporation of this covariate into our model.

“Baseline” serum creatinine was selected from *most recent* value collected within 365 days before surgery. Multiple definitions for baseline creatinine were considered, including: (i) *highest* preoperative creatinine (within the collection window) and (ii) *median* (of all values within the collection window); however, these alternatives might be biased by patients with historic renal failure who made renal recovery prior to surgery or by those with an AKI immediately before the surgery. The 365-day preoperative window was selected a priori to maximize sample size. Analysis following data collection revealed most creatinine draws occurred within the week prior to surgery, with only a limited number of cases utilizing a creatinine baseline outside of 30-days preoperatively.

The results of this initial study suggest that nongenetic risk factors (including potentially intraoperative variables) likely exert overwhelming influence on a patient’s overall AKI risk and should be a focus of ongoing research. This will require additional integration beyond the scope of this initial study but will provide even greater certainty regarding the limited contribution of genetic variants to the development of postoperative AKI when compared to other factors.

In summary, our genome-wide approach failed to identify genetic variants with genome-wide significance that predicted the risk for postoperative AKI. While larger samples or dense sequence data may help identify associated markers with small effect sizes or ones that occur at very low frequencies not detectable in our current sample, it is not clear whether such results would be useful for clinical practice; we expect that the effect of nongenetic risk factors will continue to dwarf genetic effects. Consistent with the GWAS results, we observed a poor PRS predictive ability and a very low estimated heritability for postoperative AKI. These findings suggest that genetics is unlikely to provide substantial clinical utility for reliably identifying patients at risk for postoperative AKI after noncardiac surgery, and that classification of high-risk patients based on environmental or lifestyle factors should be prioritized.

## Supplementary Information

**Additional file 1: Figure S1.** Meta-analysis of the genome-wide association results for acute kidney injury are depicted by Manhattan (1A-1C) plots. A. The model was adjusted for age, gender, and 4 principal components. B. The model was adjusted for age, gender, 4 principal components, and preoperative serum creatinine. C. The model was adjusted for age, gender, 4 principal components, preoperative serum creatinine, body mass index, preoperative hemoglobin, Elixhauser comorbidity measures, case duration, general anesthesia performed. **Figure S2.** Meta-analysis of the genome-wide association results for acute kidney injury are depicted by the quantile-quantile (QQ; 2A-2C) plots. A. The model was adjusted for age, gender, and 4 principal components. B. The model was adjusted for age, gender, 4 principal components, and preoperative serum creatinine. C. The model was adjusted for age, gender, 4 principal components, preoperative serum creatinine, body mass index, preoperative hemoglobin, Elixhauser comorbidity measures, case duration, general anesthesia performed.

## Data Availability

The data that support the findings of this study are available from corresponding author, but restrictions apply to the availability of these data, which were used under license for the current study, and so are not publicly available. Data are, however, available from the authors upon reasonable request and with permission of corresponding author.
