# Supplementary material for: Limited clinical utility for GWAS or polygenic risk score for postoperative acute kidney injury in non-cardiac surgery in European-ancestry patients
Source: BMC Nephrol. 2022 Oct 21;23:339. doi: 10.1186/s12882-022-02964-8 (PMC9587619; doi:10.1186/s12882-022-02964-8)
Supplement: Supplementary file 1 — Additional file 1: Figure S1. Meta-analysis of the genome-wide association results for acute kidney injury are depicted by Manhattan (1A-1C) plots. A. The model was adjusted for age, gender, and 4 principal components. B. The model was adjusted for age, gender, 4 principal components, and preoperative serum creatinine. C. The model was adjusted for age, gender, 4 principal components, preoperative serum creatinine, body mass index, preoperative hemoglobin, Elixhauser comorbidity measures, case duration, general anesthesia performed. Figure S2. Meta-analysis of the genome-wide association results for acute kidney injury are depicted by the quantile-quantile (QQ; 2A-2C) plots. A. The model was adjusted for age, gender, and 4 principal components. B. The model was adjusted for age, gender, 4 principal components, and preoperative serum creatinine. C. The model was adjusted for age, gender, 4 principal components, preoperative serum creatinine, body mass index, preoperative hemoglobin, Elixhauser comorbidity measures, case duration, general anesthesia performed. [file 12882_2022_2964_MOESM1_ESM.docx]

**SUPPLEMENTARY MATERIALS**

**SUPPLEMENTARY METHODS**

**Genetic Data**

As part of the independent data flow for both individual sites, sample and genotype quality control included assessment of call rates, gender check, cryptic relatedness, SNP missingness and the Hardy-Weinberg equilibrium.^29^ Principal components (PCs) were generated using the EIGENSTRAT method in EigenSoft version 7.2.1. program to control for population stratification.^30^

Imputation of the Vanderbilt MEGA^EX^ genotyped dataset was performed using the Minimac4 and 1000 Genomes Phase 3 v5 with the Michigan Imputation Server.^31^ Variants with R^2^ > 0.3 were selected, resulting in more than 21.1 million overall imputed variants available and 11.7 million imputed variants available for the AKI GWAS with White European ancestry after quality control and filtering. Imputation of the Michigan CoreExome Array genotyped data was performed using the Haplotype Reference Consortium with the Michigan Imputation Server.^31^ Again, variants with R^2^ > 0.3 and minor allele frequency (MAF) > 0.01% were selected, resulting in more than 17 million imputed variants with White European ancestry after quality control and filtering. This resulted in 6.95 million overlapping variants between the Vanderbilt and Michigan cohorts for the postoperative AKI meta-analysis with White European Ancestry.

**SUPPLEMENTARY RESULTS**

**Figure S1.** Meta-analysis of the genome wide association results for acute kidney injury are depicted by Manhattan (1A-1C) plots.

**Figure S1A.** The model was adjusted for age, gender, and 4 principal components


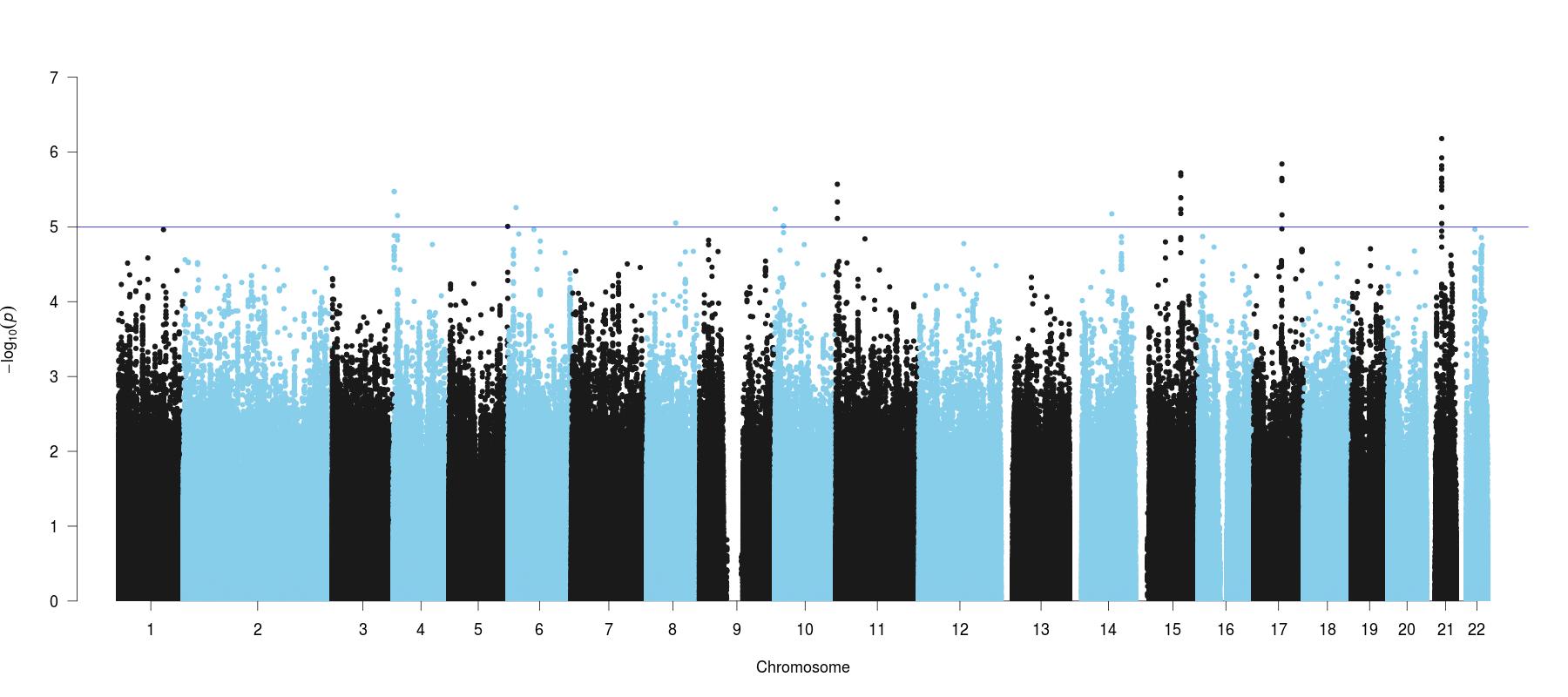


**Figure S1B.** The model was adjusted for age, gender, 4 principal components, and preoperative serum creatinine


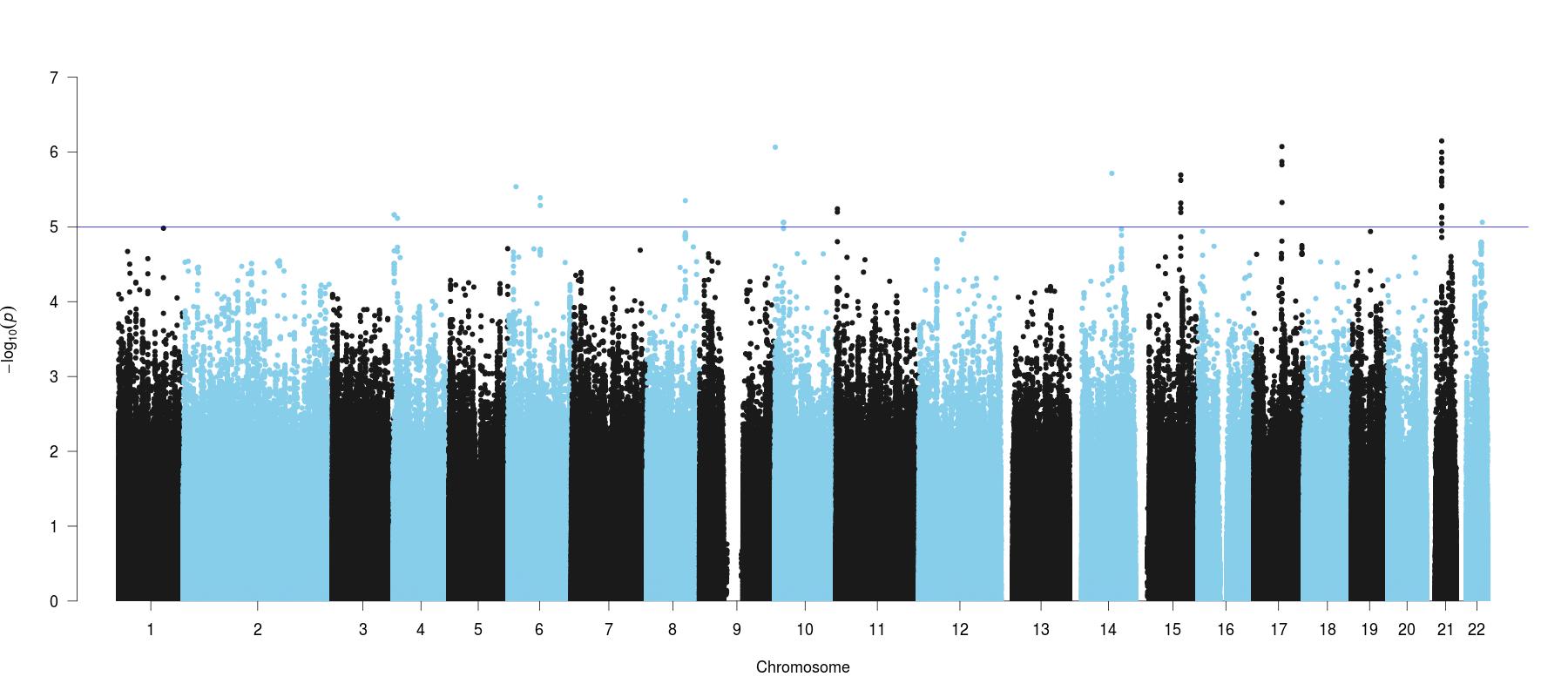


**Figure S1C.** The model was adjusted for age, gender, 4 principal components, preoperative serum creatinine, body mass index, preoperative hemoglobin, Elixhauser comorbidity measures, case duration, general anesthesia performed


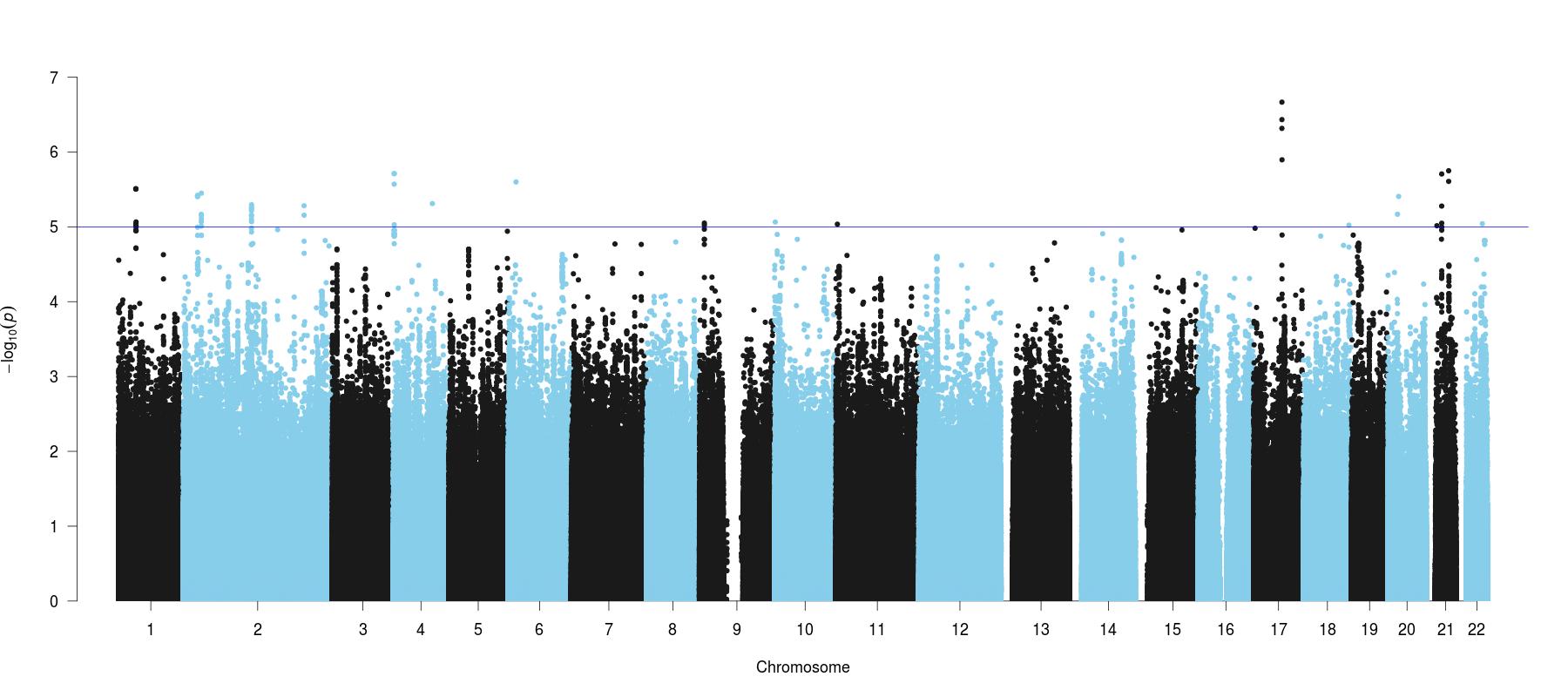


**Figure S2.** Meta-analysis of the genome wide association results for acute kidney injury are depicted by the quantile-quantile (QQ; 2A-2C) plots.

**Figure S2A.** The model was adjusted for age, gender, and 4 principal components

**
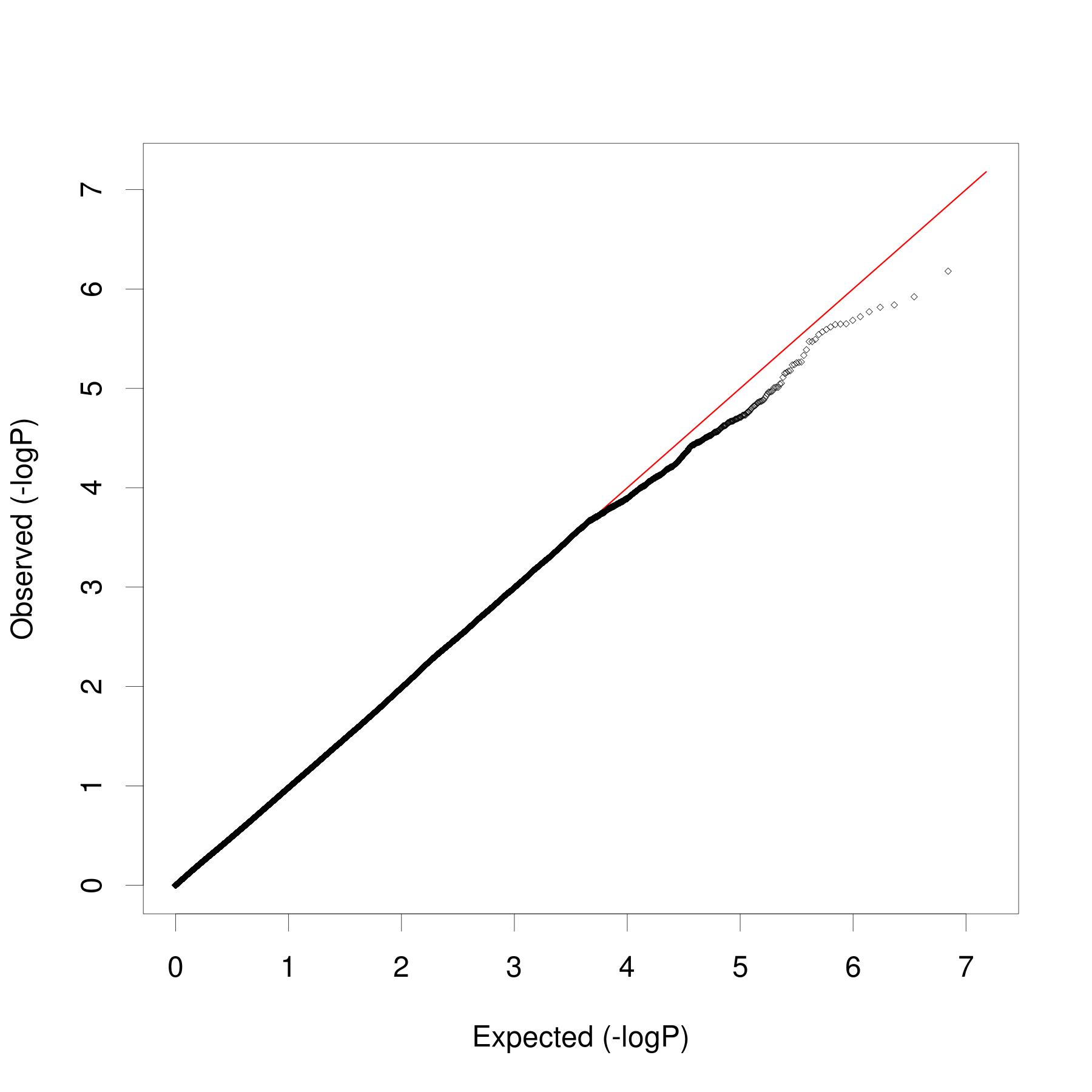
**

**Figure S2B.** The model was adjusted for age, gender, 4 principal components, and preoperative serum creatinine

**
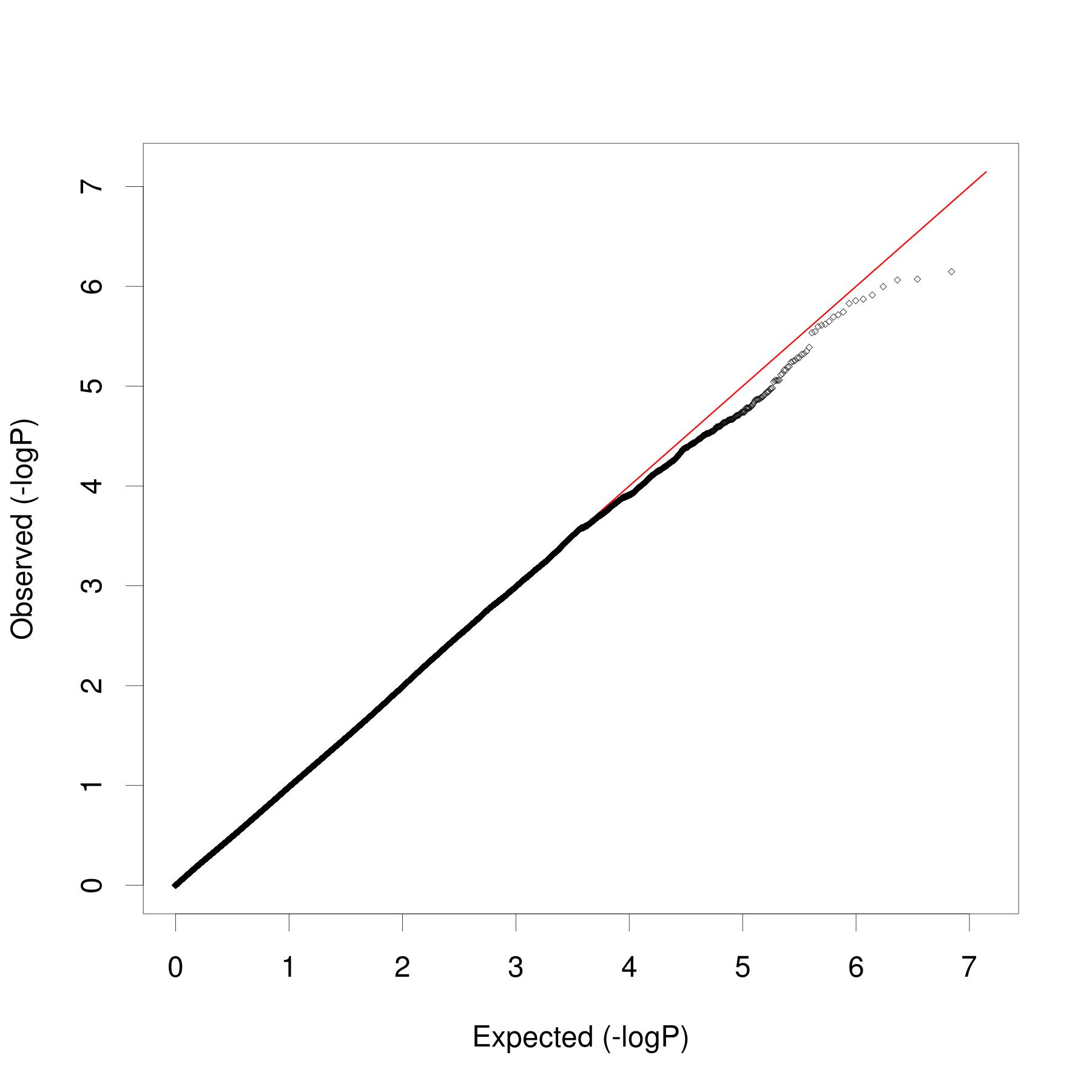
**

**Figure S2C.** The model was adjusted for age, gender, 4 principal components, preoperative serum creatinine, body mass index, preoperative hemoglobin, Elixhauser comorbidity measures, case duration, general anesthesia performed

**
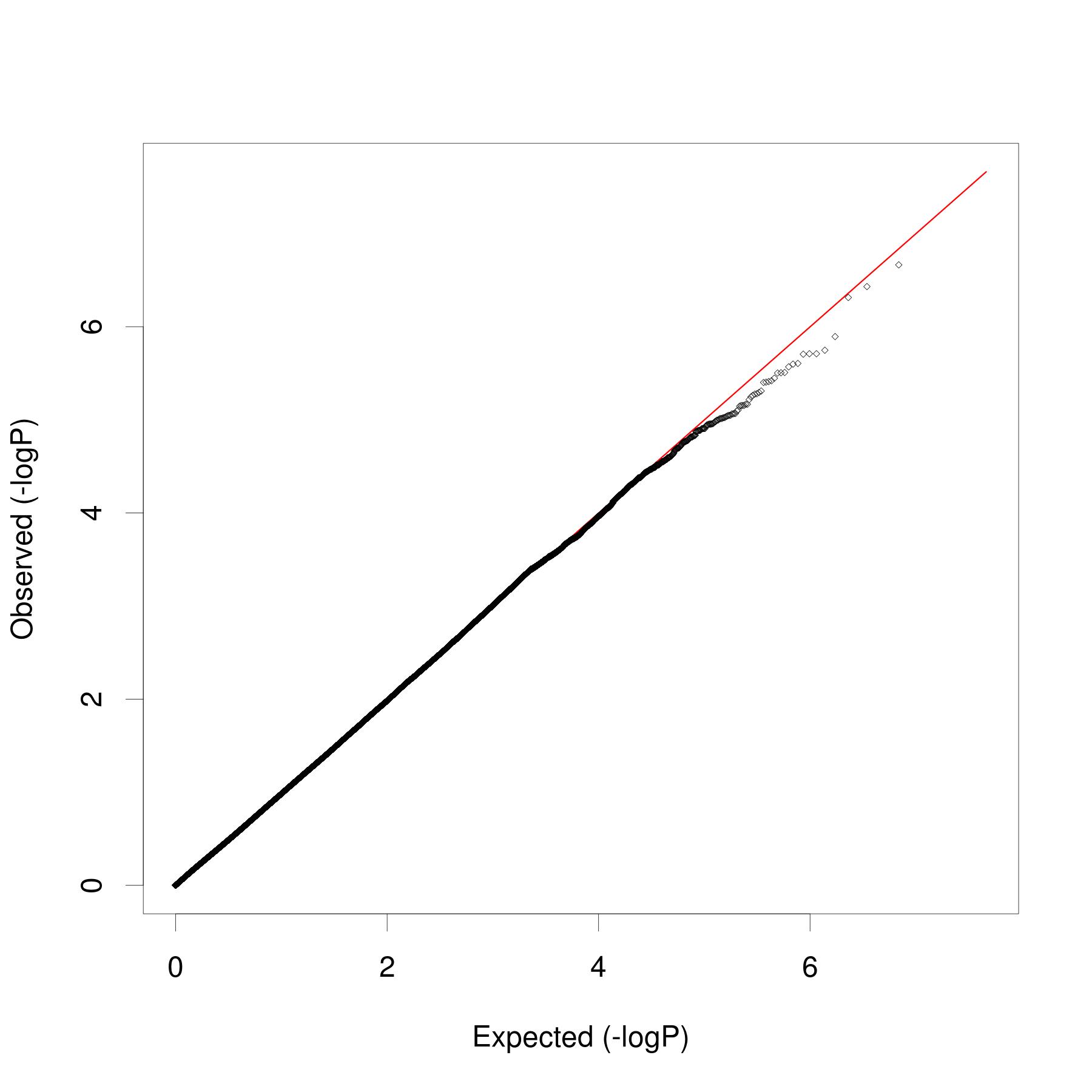
**
